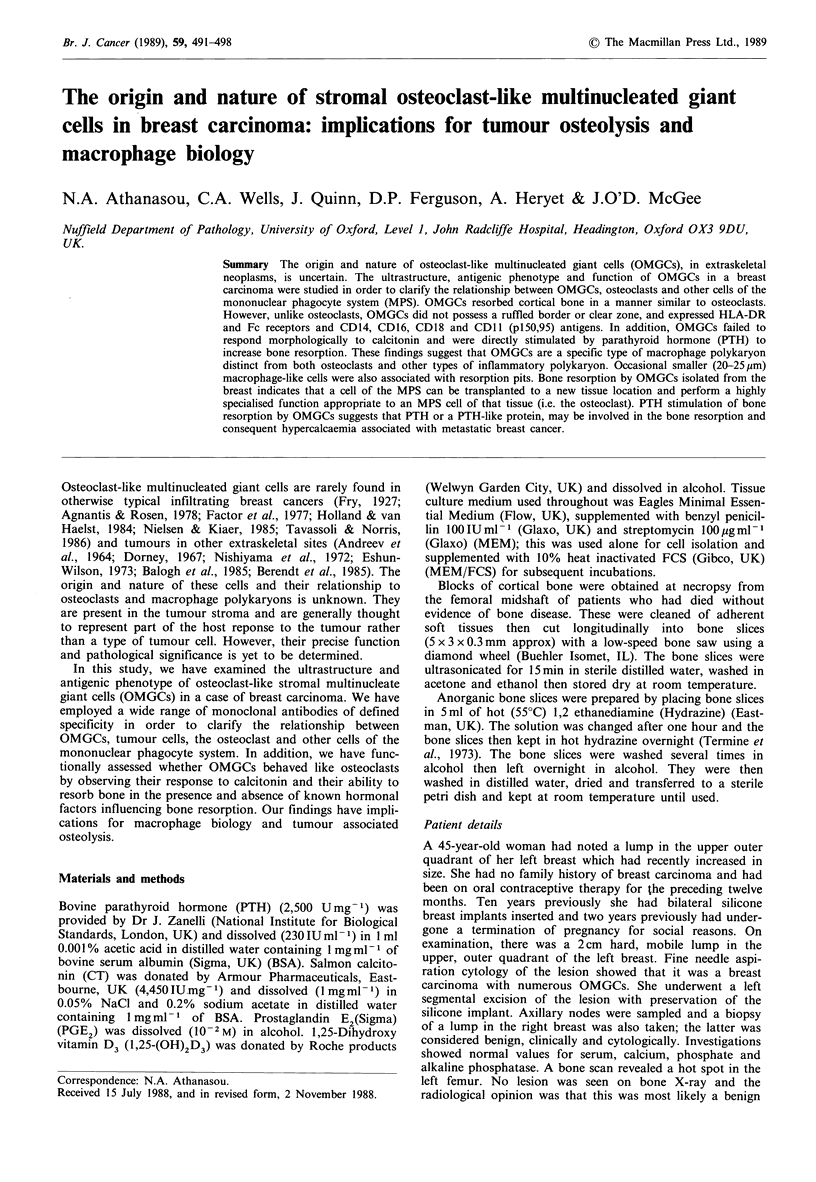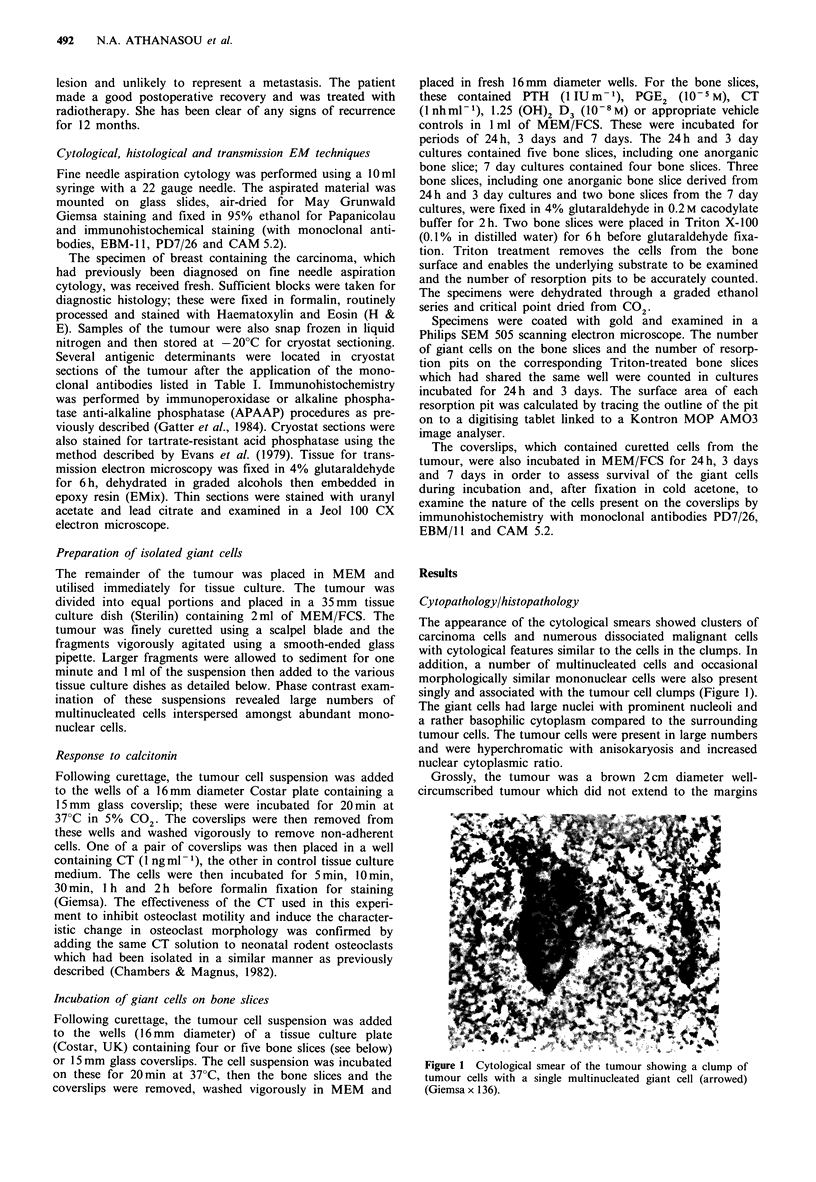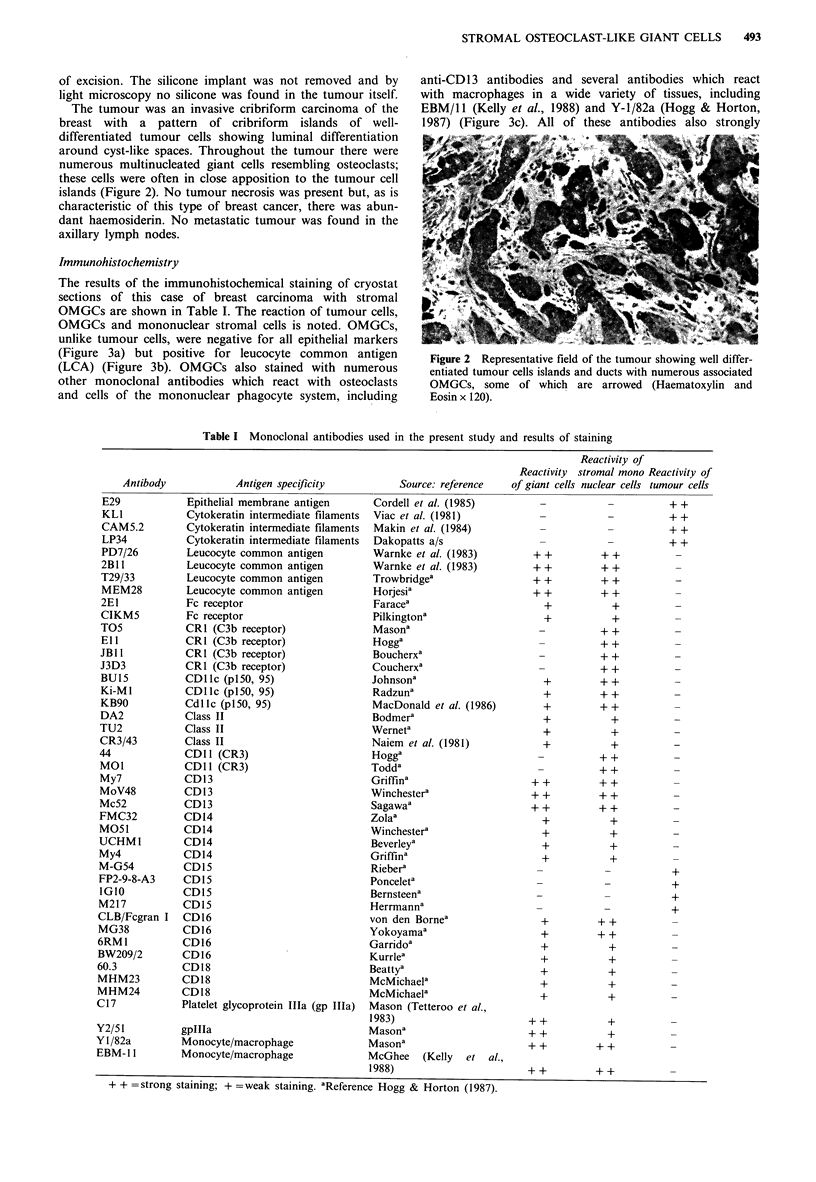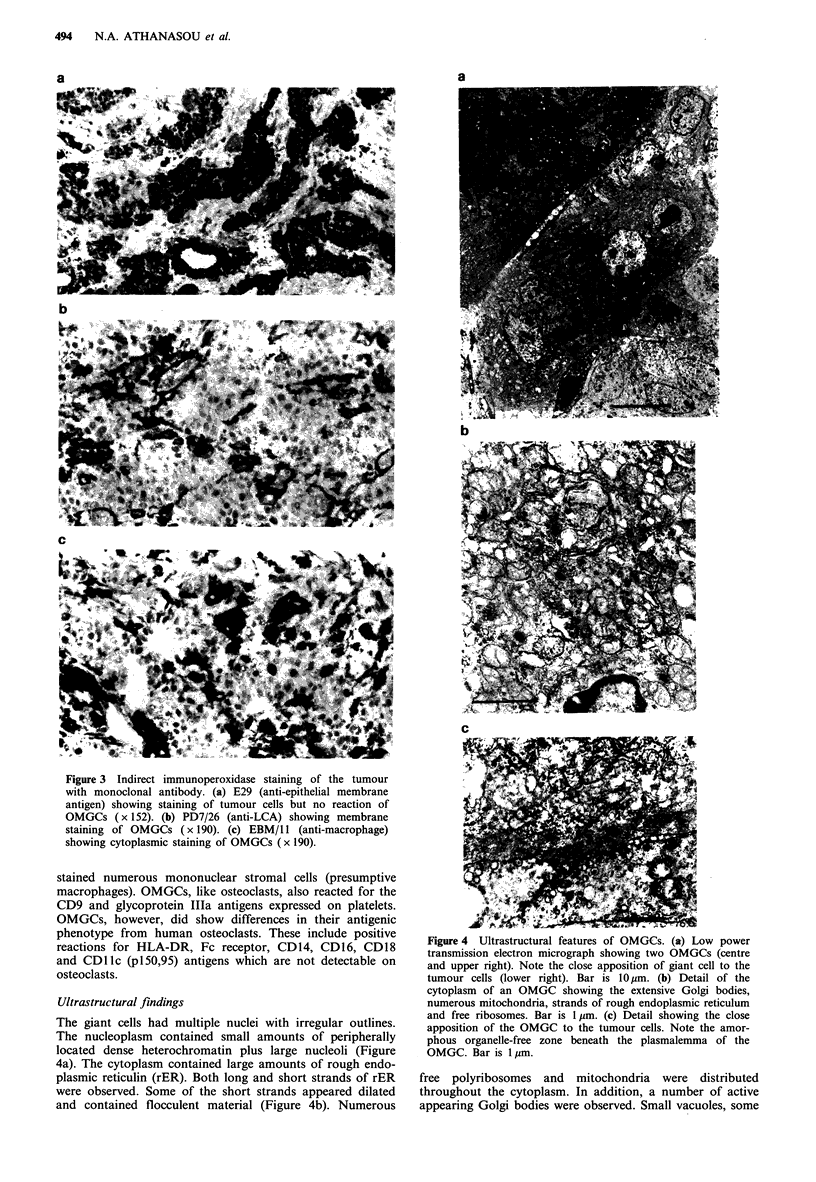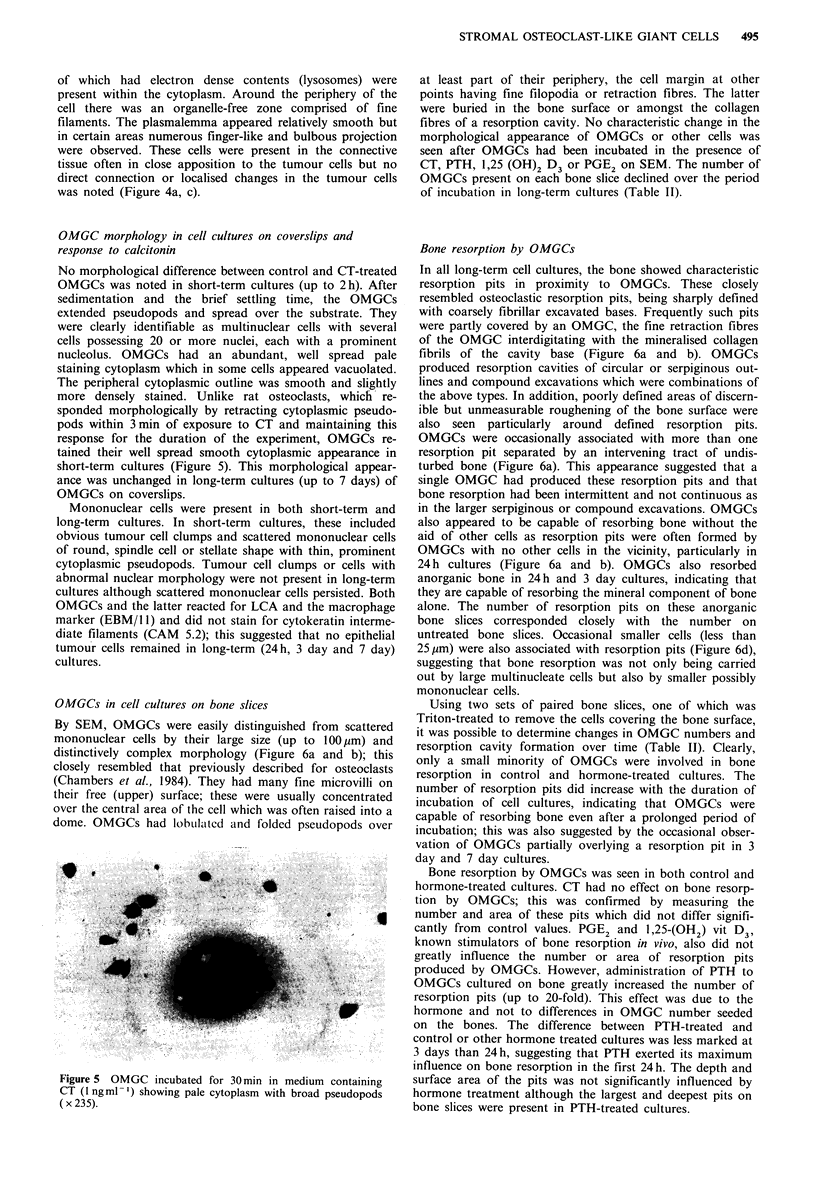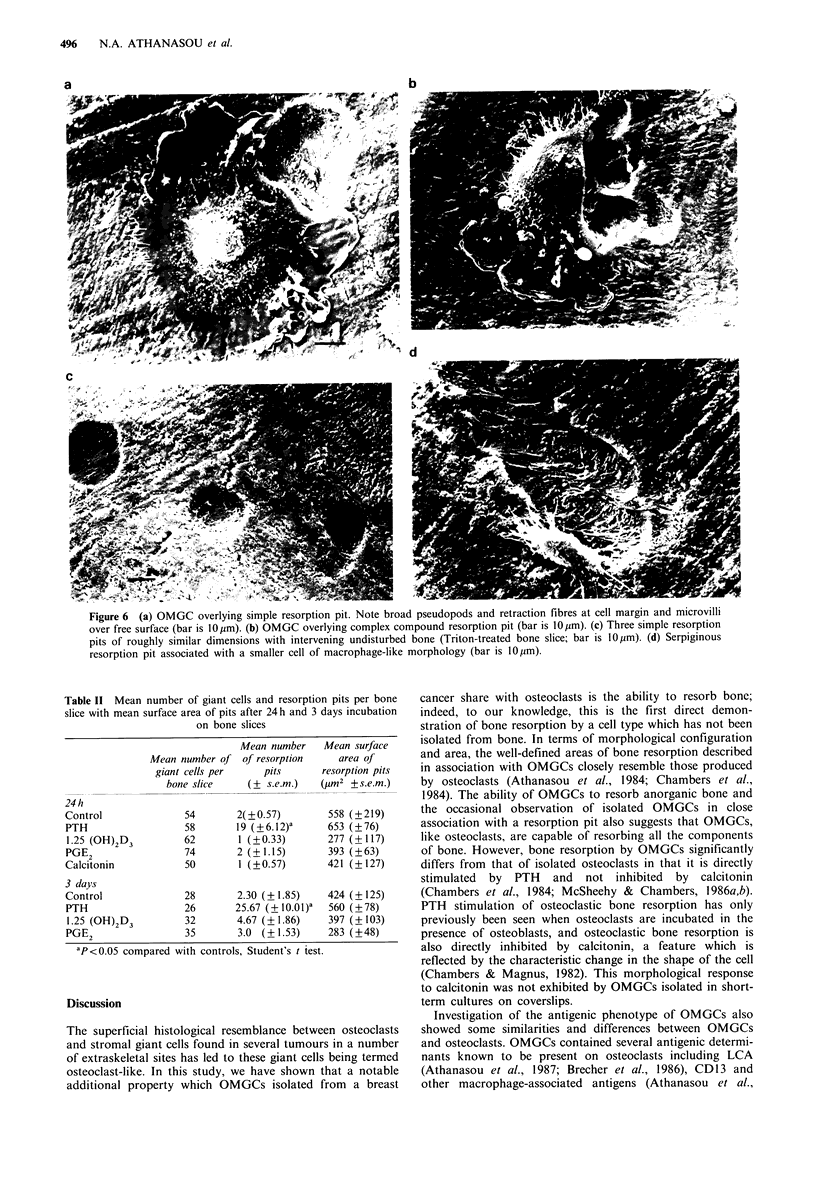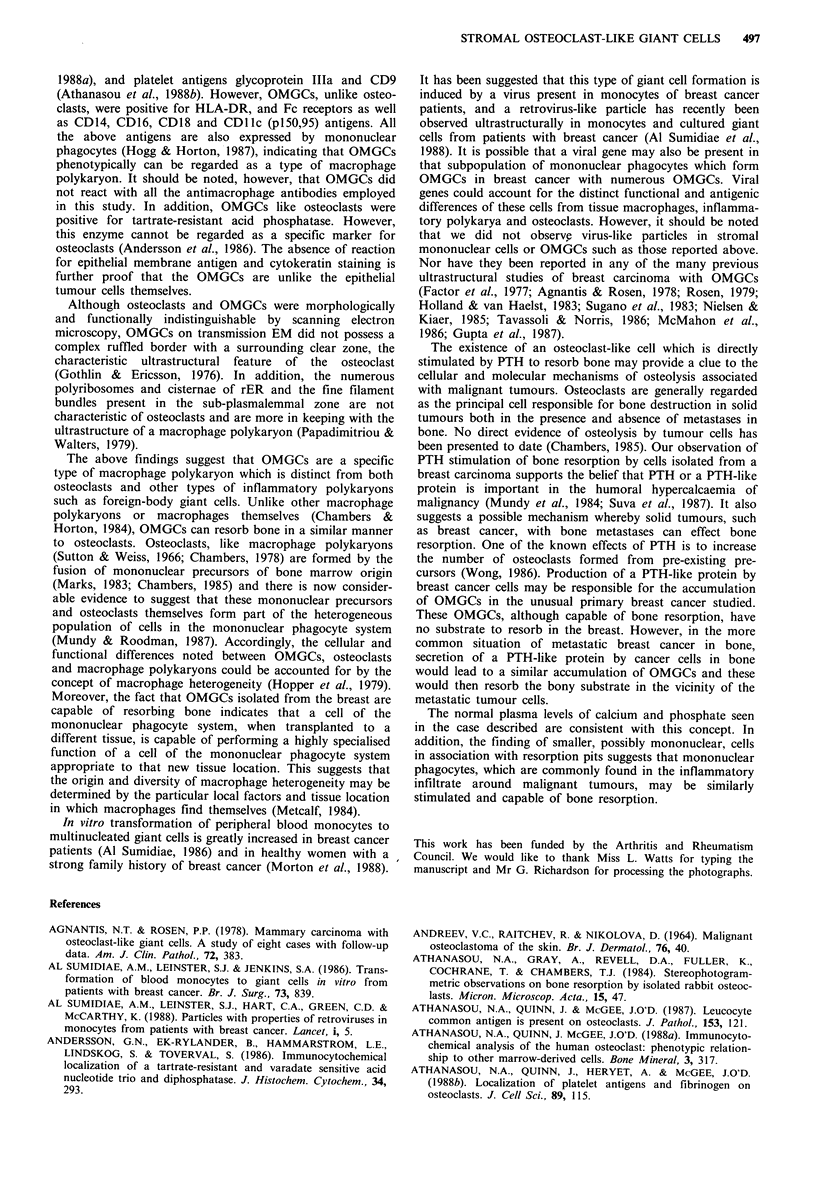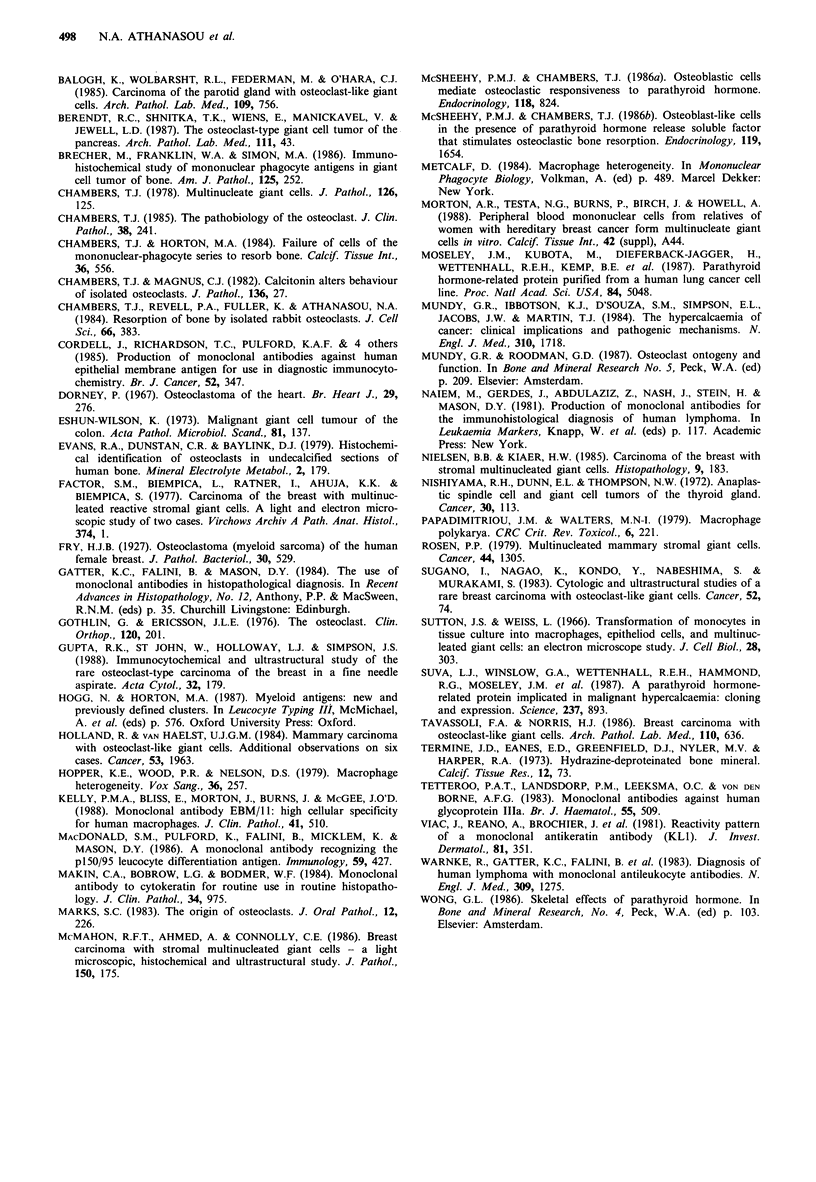# The origin and nature of stromal osteoclast-like multinucleated giant cells in breast carcinoma: implications for tumour osteolysis and macrophage biology.

**DOI:** 10.1038/bjc.1989.102

**Published:** 1989-04

**Authors:** N. A. Athanasou, C. A. Wells, J. Quinn, D. P. Ferguson, A. Heryet, J. O. McGee

**Affiliations:** Nuffield Department of Pathology, University of Oxford, John Radcliffe Hospital, Headington, UK.

## Abstract

**Images:**


*Br. J. Cancer* (1989), **59**, 491–498 © The Macmillan Press Ltd., 1989

### The origin and nature of stromal osteoclast-like multinucleated giant cells in breast carcinoma: implications for tumour osteolysis and macrophage biology

N.A. Athanasou, C.A. Wells, J. Quinn, D.P. Ferguson, A. Heryet & J.O'D. McGee

*Nuffield Department of Pathology, University of Oxford, Level 1, John Radcliffe Hospital, Headington, Oxford OX3 9DU, UK.*

**Summary** The origin and nature of osteoclast-like multinucleated giant cells (OMGCs), in extraskeletal neoplasms, is uncertain. The ultrastructure, antigenic phenotype and function of OMGCs in a breast carcinoma were studied in order to clarify the relationship between OMGCs, osteoclasts and other cells of the mononuclear phagocyte system (MPS). OMGCs resorbed cortical bone in a manner similar to osteoclasts. However, unlike osteoclasts, OMGCs did not possess a ruffled border or clear zone, and expressed HLA-DR and Fc receptors and CD14, CD16, CD18 and CD11 (p150,95) antigens. In addition, OMGCs failed to respond morphologically to calcitonin and were directly stimulated by parathyroid hormone (PTH) to increase bone resorption. These findings suggest that OMGCs are a specific type of macrophage polykaryon distinct from both osteoclasts and other types of inflammatory polykaryon. Occasional smaller (20–25 $\mu$m) macrophage-like cells were also associated with resorption pits. Bone resorption by OMGCs isolated from the breast indicates that a cell of the MPS can be transplanted to a new tissue location and perform a highly specialised function appropriate to an MPS cell of that tissue (i.e. the osteoclast). PTH stimulation of bone resorption by OMGCs suggests that PTH or a PTH-like protein, may be involved in the bone resorption and consequent hypercalcaemia associated with metastatic breast cancer.

Osteoclast-like multinucleated giant cells are rarely found in otherwise typical infiltrating breast cancers (Fry, 1927; Agnantis & Rosen, 1978; Factor *et al.*, 1977; Holland & van Haelst, 1984; Nielsen & Kiaer, 1985; Tavassoli & Norris, 1986) and tumours in other extraskeletal sites (Andreev *et al.*, 1964; Dorney, 1967; Nishiyama *et al.*, 1972; Eshun-Wilson, 1973; Balogh *et al.*, 1985; Berendt *et al.*, 1985). The origin and nature of these cells and their relationship to osteoclasts and macrophage polykaryons is unknown. They are present in the tumour stroma and are generally thought to represent part of the host reponse to the tumour rather than a type of tumour cell. However, their precise function and pathological significance is yet to be determined.

In this study, we have examined the ultrastructure and antigenic phenotype of osteoclast-like stromal multinucleate giant cells (OMGCs) in a case of breast carcinoma. We have employed a wide range of monoclonal antibodies of defined specificity in order to clarify the relationship between OMGCs, tumour cells, the osteoclast and other cells of the mononuclear phagocyte system. In addition, we have functionally assessed whether OMGCs behaved like osteoclasts by observing their response to calcitonin and their ability to resorb bone in the presence and absence of known hormonal factors influencing bone resorption. Our findings have implications for macrophage biology and tumour associated osteolysis.

### Materials and methods

Bovine parathyroid hormone (PTH) (2,500 U mg$^{-1}$) was provided by Dr J. Zanelli (National Institute for Biological Standards, London, UK) and dissolved (230 IU ml$^{-1}$) in 1 ml 0.001% acetic acid in distilled water containing 1 mg ml$^{-1}$ of bovine serum albumin (Sigma, UK) (BSA). Salmon calcitonin (CT) was donated by Armour Pharmaceuticals, Eastbourne, UK (4,450 IU mg$^{-1}$) and dissolved (1 mg ml$^{-1}$) in 0.05% NaCl and 0.2% sodium acetate in distilled water containing 1 mg ml$^{-1}$ of BSA. Prostaglandin $E_2$(Sigma) ($PGE_2$) was dissolved ($10^{-2}$ M) in alcohol. 1,25-Dihydroxy vitamin $D_3$ (1,25-$(OH)_2D_3$) was donated by Roche products (Welwyn Garden City, UK) and dissolved in alcohol. Tissue culture medium used throughout was Eagles Minimal Essential Medium (Flow, UK), supplemented with benzyl penicillin 100 IU ml$^{-1}$ (Glaxo, UK) and streptomycin 100 $\mu$g ml$^{-1}$ (Glaxo) (MEM); this was used alone for cell isolation and supplemented with 10% heat inactivated FCS (Gibco, UK) (MEM/FCS) for subsequent incubations.

Blocks of cortical bone were obtained at necropsy from the femoral midshaft of patients who had died without evidence of bone disease. These were cleaned of adherent soft tissues then cut longitudinally into bone slices (5 x 3 x 0.3 mm approx) with a low-speed bone saw using a diamond wheel (Buehler Isomet, IL). The bone slices were ultrasonicated for 15 min in sterile distilled water, washed in acetone and ethanol then stored dry at room temperature.

Anorganic bone slices were prepared by placing bone slices in 5 ml of hot (55°C) 1,2 ethanediamine (Hydrazine) (Eastman, UK). The solution was changed after one hour and the bone slices then kept in hot hydrazine overnight (Termine *et al.*, 1973). The bone slices were washed several times in alcohol then left overnight in alcohol. They were then washed in distilled water, dried and transferred to a sterile petri dish and kept at room temperature until used.

### *Patient details*

A 45-year-old woman had noted a lump in the upper outer quadrant of her left breast which had recently increased in size. She had no family history of breast carcinoma and had been on oral contraceptive therapy for the preceding twelve months. Ten years previously she had bilateral silicone breast implants inserted and two years previously had undergone a termination of pregnancy for social reasons. On examination, there was a 2 cm hard, mobile lump in the upper, outer quadrant of the left breast. Fine needle aspiration cytology of the lesion showed that it was a breast carcinoma with numerous OMGCs. She underwent a left segmental excision of the lesion with preservation of the silicone implant. Axillary nodes were sampled and a biopsy of a lump in the right breast was also taken; the latter was considered benign, clinically and cytologically. Investigations showed normal values for serum, calcium, phosphate and alkaline phosphatase. A bone scan revealed a hot spot in the left femur. No lesion was seen on bone X-ray and the radiological opinion was that this was most likely a benign

Correspondence: N.A. Athanasou.
Received 15 July 1988, and in revised form, 2 November 1988.

lesion and unlikely to represent a metastasis. The patient made a good postoperative recovery and was treated with radiotherapy. She has been clear of any signs of recurrence for 12 months.

*Cytological, histological and transmission EM techniques*

Fine needle aspiration cytology was performed using a 10 ml syringe with a 22 gauge needle. The aspirated material was mounted on glass slides, air-dried for May Grunwald Giemsa staining and fixed in 95% ethanol for Papanicolau and immunohistochemical staining (with monoclonal antibodies, EBM-11, PD7/26 and CAM 5.2).

The specimen of breast containing the carcinoma, which had previously been diagnosed on fine needle aspiration cytology, was received fresh. Sufficient blocks were taken for diagnostic histology; these were fixed in formalin, routinely processed and stained with Haematoxylin and Eosin (H & E). Samples of the tumour were also snap frozen in liquid nitrogen and then stored at $-20°C$ for cryostat sectioning. Several antigenic determinants were located in cryostat sections of the tumour after the application of the monoclonal antibodies listed in Table I. Immunohistochemistry was performed by immunoperoxidase or alkaline phosphatase anti-alkaline phosphatase (APAAP) procedures as previously described (Gatter *et al.*, 1984). Cryostat sections were also stained for tartrate-resistant acid phosphatase using the method described by Evans *et al.* (1979). Tissue for transmission electron microscopy was fixed in 4% glutaraldehyde for 6 h, dehydrated in graded alcohols then embedded in epoxy resin (EMix). Thin sections were stained with uranyl acetate and lead citrate and examined in a Jeol 100 CX electron microscope.

*Preparation of isolated giant cells*

The remainder of the tumour was placed in MEM and utilised immediately for tissue culture. The tumour was divided into equal portions and placed in a 35 mm tissue culture dish (Sterilin) containing 2 ml of MEM/FCS. The tumour was finely curetted using a scalpel blade and the fragments vigorously agitated using a smooth-ended glass pipette. Larger fragments were allowed to sediment for one minute and 1 ml of the suspension then added to the various tissue culture dishes as detailed below. Phase contrast examination of these suspensions revealed large numbers of multinucleated cells interspersed amongst abundant mononuclear cells.

*Response to calcitonin*

Following curettage, the tumour cell suspension was added to the wells of a 16 mm diameter Costar plate containing a 15 mm glass coverslip; these were incubated for 20 min at 37°C in 5% $CO_2$. The coverslips were then removed from these wells and washed vigorously to remove non-adherent cells. One of a pair of coverslips was then placed in a well containing CT ($1\ ng\ ml^{-1}$), the other in control tissue culture medium. The cells were then incubated for 5 min, 10 min, 30 min, 1 h and 2 h before formalin fixation for staining (Giemsa). The effectiveness of the CT used in this experiment to inhibit osteoclast motility and induce the characteristic change in osteoclast morphology was confirmed by adding the same CT solution to neonatal rodent osteoclasts which had been isolated in a similar manner as previously described (Chambers & Magnus, 1982).

*Incubation of giant cells on bone slices*

Following curettage, the tumour cell suspension was added to the wells (16 mm diameter) of a tissue culture plate (Costar, UK) containing four or five bone slices (see below) or 15 mm glass coverslips. The cell suspension was incubated on these for 20 min at 37°C, then the bone slices and the coverslips were removed, washed vigorously in MEM and placed in fresh 16 mm diameter wells. For the bone slices, these contained PTH ($1\ IU\ m^{-1}$), $PGE_2$ ($10^{-5}$ M), CT ($1\ nh\ ml^{-1}$), 1.25 $(OH)_2\ D_3$ ($10^{-8}$ M) or appropriate vehicle controls in 1 ml of MEM/FCS. These were incubated for periods of 24 h, 3 days and 7 days. The 24 h and 3 day cultures contained five bone slices, including one anorganic bone slice; 7 day cultures contained four bone slices. Three bone slices, including one anorganic bone slice derived from 24 h and 3 day cultures and two bone slices from the 7 day cultures, were fixed in 4% glutaraldehyde in 0.2 M cacodylate buffer for 2 h. Two bone slices were placed in Triton X-100 (0.1% in distilled water) for 6 h before glutaraldehyde fixation. Triton treatment removes the cells from the bone surface and enables the underlying substrate to be examined and the number of resorption pits to be accurately counted. The specimens were dehydrated through a graded ethanol series and critical point dried from $CO_2$.

Specimens were coated with gold and examined in a Philips SEM 505 scanning electron microscope. The number of giant cells on the bone slices and the number of resorption pits on the corresponding Triton-treated bone slices which had shared the same well were counted in cultures incubated for 24 h and 3 days. The surface area of each resorption pit was calculated by tracing the outline of the pit on to a digitising tablet linked to a Kontron MOP AMO3 image analyser.

The coverslips, which contained curetted cells from the tumour, were also incubated in MEM/FCS for 24 h, 3 days and 7 days in order to assess survival of the giant cells during incubation and, after fixation in cold acetone, to examine the nature of the cells present on the coverslips by immunohistochemistry with monoclonal antibodies PD7/26, EBM/11 and CAM 5.2.

### Results

*Cytopathology/histopathology*

The appearance of the cytological smears showed clusters of carcinoma cells and numerous dissociated malignant cells with cytological features similar to the cells in the clumps. In addition, a number of multinucleated cells and occasional morphologically similar mononuclear cells were also present singly and associated with the tumour cell clumps (Figure 1). The giant cells had large nuclei with prominent nucleoli and a rather basophilic cytoplasm compared to the surrounding tumour cells. The tumour cells were present in large numbers and were hyperchromatic with anisokaryosis and increased nuclear cytoplasmic ratio.

Grossly, the tumour was a brown 2 cm diameter well-circumscribed tumour which did not extend to the margins

**Figure 1** Cytological smear of the tumour showing a clump of tumour cells with a single multinucleated giant cell (arrowed) (Giemsa × 136).

of excision. The silicone implant was not removed and by light microscopy no silicone was found in the tumour itself.

The tumour was an invasive cribriform carcinoma of the breast with a pattern of cribriform islands of well-differentiated tumour cells showing luminal differentiation around cyst-like spaces. Throughout the tumour there were numerous multinucleated giant cells resembling osteoclasts; these cells were often in close apposition to the tumour cell islands (Figure 2). No tumour necrosis was present but, as is characteristic of this type of breast cancer, there was abundant haemosiderin. No metastatic tumour was found in the axillary lymph nodes.

*Immunohistochemistry*

The results of the immunohistochemical staining of cryostat sections of this case of breast carcinoma with stromal OMGCs are shown in Table I. The reaction of tumour cells, OMGCs and mononuclear stromal cells is noted. OMGCs, unlike tumour cells, were negative for all epithelial markers (Figure 3a) but positive for leucocyte common antigen (LCA) (Figure 3b). OMGCs also stained with numerous other monoclonal antibodies which react with osteoclasts and cells of the mononuclear phagocyte system, including anti-CD13 antibodies and several antibodies which react with macrophages in a wide variety of tissues, including EBM/11 (Kelly *et al.*, 1988) and Y-1/82a (Hogg & Horton, 1987) (Figure 3c). All of these antibodies also strongly

**Figure 2** Representative field of the tumour showing well differentiated tumour cells islands and ducts with numerous associated OMGCs, some of which are arrowed (Haematoxylin and Eosin x 120).

**Table I** Monoclonal antibodies used in the present study and results of staining

| *Antibody* | *Antigen specificity* | *Source: reference* | *Reactivity of giant cells* | *Reactivity of stromal mono nuclear cells* | *Reactivity of tumour cells* |
|---|---|---|---|---|---|
| E29 | Epithelial membrane antigen | Cordell *et al.* (1985) | – | – | ++ |
| KL1 | Cytokeratin intermediate filaments | Viac *et al.* (1981) | – | – | ++ |
| CAM5.2 | Cytokeratin intermediate filaments | Makin *et al.* (1984) | – | – | ++ |
| LP34 | Cytokeratin intermediate filaments | Dakopatts a/s | – | – | ++ |
| PD7/26 | Leucocyte common antigen | Warnke *et al.* (1983) | ++ | ++ | – |
| 2B11 | Leucocyte common antigen | Warnke *et al.* (1983) | ++ | ++ | – |
| T29/33 | Leucocyte common antigen | Trowbridge[a] | ++ | ++ | – |
| MEM28 | Leucocyte common antigen | Horjesi[a] | ++ | ++ | – |
| 2E1 | Fc receptor | Farace[a] | + | + | – |
| CIKM5 | Fc receptor | Pilkington[a] | + | + | – |
| TO5 | CR1 (C3b receptor) | Mason[a] | – | ++ | – |
| E11 | CR1 (C3b receptor) | Hogg[a] | – | ++ | – |
| JB11 | CR1 (C3b receptor) | Boucherx[a] | – | ++ | – |
| J3D3 | CR1 (C3b receptor) | Coucherx[a] | – | ++ | – |
| BU15 | CD11c (p150, 95) | Johnson[a] | + | ++ | – |
| Ki-M1 | CD11c (p150, 95) | Radzun[a] | + | ++ | – |
| KB90 | Cd11c (p150, 95) | MacDonald *et al.* (1986) | + | ++ | – |
| DA2 | Class II | Bodmer[a] | + | + | – |
| TU2 | Class II | Wernet[a] | + | + | – |
| CR3/43 | Class II | Naiem *et al.* (1981) | + | + | – |
| 44 | CD11 (CR3) | Hogg[a] | – | ++ | – |
| MO1 | CD11 (CR3) | Todd[a] | – | ++ | – |
| My7 | CD13 | Griffin[a] | ++ | ++ | – |
| MoV48 | CD13 | Winchester[a] | ++ | ++ | – |
| Mc52 | CD13 | Sagawa[a] | ++ | ++ | – |
| FMC32 | CD14 | Zola[a] | + | + | – |
| MO51 | CD14 | Winchester[a] | + | + | – |
| UCHM1 | CD14 | Beverley[a] | + | + | – |
| My4 | CD14 | Griffin[a] | + | + | – |
| M-G54 | CD15 | Rieber[a] | – | – | + |
| FP2-9-8-A3 | CD15 | Poncelet[a] | – | – | + |
| 1G10 | CD15 | Bernsteen[a] | – | – | + |
| M217 | CD15 | Herrmann[a] | – | – | + |
| CLB/Fcgran I | CD16 | von den Borne[a] | + | ++ | – |
| MG38 | CD16 | Yokoyama[a] | + | ++ | – |
| 6RM1 | CD16 | Garrido[a] | + | + | – |
| BW209/2 | CD16 | Kurrle[a] | + | + | – |
| 60.3 | CD18 | Beatty[a] | + | + | – |
| MHM23 | CD18 | McMichael[a] | + | + | – |
| MHM24 | CD18 | McMichael[a] | + | + | – |
| C17 | Platelet glycoprotein IIIa (gp IIIa) | Mason (Tetteroo *et al.*, 1983) | ++ | + | – |
| Y2/51 | gpIIIa | Mason[a] | ++ | + | – |
| Y1/82a | Monocyte/macrophage | Mason[a] | ++ | ++ | – |
| EBM-11 | Monocyte/macrophage | McGhee (Kelly *et al.*, 1988) | ++ | ++ | – |

++ = strong staining; + = weak staining. [a]Reference Hogg & Horton (1987).

**Figure 3** Indirect immunoperoxidase staining of the tumour with monoclonal antibody. (**a**) E29 (anti-epithelial membrane antigen) showing staining of tumour cells but no reaction of OMGCs (×152). (**b**) PD7/26 (anti-LCA) showing membrane staining of OMGCs (×190). (**c**) EBM/11 (anti-macrophage) showing cytoplasmic staining of OMGCs (×190).

stained numerous mononuclear stromal cells (presumptive macrophages). OMGCs, like osteoclasts, also reacted for the CD9 and glycoprotein IIIa antigens expressed on platelets. OMGCs, however, did show differences in their antigenic phenotype from human osteoclasts. These include positive reactions for HLA-DR, Fc receptor, CD14, CD16, CD18 and CD11c (p150,95) antigens which are not detectable on osteoclasts.

*Ultrastructural findings*

The giant cells had multiple nuclei with irregular outlines. The nucleoplasm contained small amounts of peripherally located dense heterochromatin plus large nucleoli (Figure 4a). The cytoplasm contained large amounts of rough endoplasmic reticulin (rER). Both long and short strands of rER were observed. Some of the short strands appeared dilated and contained flocculent material (Figure 4b). Numerous

**Figure 4** Ultrastructural features of OMGCs. (**a**) Low power transmission electron micrograph showing two OMGCs (centre and upper right). Note the close apposition of giant cell to the tumour cells (lower right). Bar is 10 μm. (**b**) Detail of the cytoplasm of an OMGC showing the extensive Golgi bodies, numerous mitochondria, strands of rough endoplasmic reticulum and free ribosomes. Bar is 1 μm. (**c**) Detail showing the close apposition of the OMGC to the tumour cells. Note the amorphous organelle-free zone beneath the plasmalemma of the OMGC. Bar is 1 μm.

free polyribosomes and mitochondria were distributed throughout the cytoplasm. In addition, a number of active appearing Golgi bodies were observed. Small vacuoles, some of which had electron dense contents (lysosomes) were present within the cytoplasm. Around the periphery of the cell there was an organelle-free zone comprised of fine filaments. The plasmalemma appeared relatively smooth but in certain areas numerous finger-like and bulbous projection were observed. These cells were present in the connective tissue often in close apposition to the tumour cells but no direct connection or localised changes in the tumour cells was noted (Figure 4a, c).

### *OMGC morphology in cell cultures on coverslips and response to calcitonin*

No morphological difference between control and CT-treated OMGCs was noted in short-term cultures (up to 2 h). After sedimentation and the brief settling time, the OMGCs extended pseudopods and spread over the substrate. They were clearly identifiable as multinuclear cells with several cells possessing 20 or more nuclei, each with a prominent nucleolus. OMGCs had an abundant, well spread pale staining cytoplasm which in some cells appeared vacuolated. The peripheral cytoplasmic outline was smooth and slightly more densely stained. Unlike rat osteoclasts, which responded morphologically by retracting cytoplasmic pseudopods within 3 min of exposure to CT and maintaining this response for the duration of the experiment, OMGCs retained their well spread smooth cytoplasmic appearance in short-term cultures (Figure 5). This morphological appearance was unchanged in long-term cultures (up to 7 days) of OMGCs on coverslips.

Mononuclear cells were present in both short-term and long-term cultures. In short-term cultures, these included obvious tumour cell clumps and scattered mononuclear cells of round, spindle cell or stellate shape with thin, prominent cytoplasmic pseudopods. Tumour cell clumps or cells with abnormal nuclear morphology were not present in long-term cultures although scattered mononuclear cells persisted. Both OMGCs and the latter reacted for LCA and the macrophage marker (EBM/11) and did not stain for cytokeratin intermediate filaments (CAM 5.2); this suggested that no epithelial tumour cells remained in long-term (24 h, 3 day and 7 day) cultures.

### *OMGCs in cell cultures on bone slices*

By SEM, OMGCs were easily distinguished from scattered mononuclear cells by their large size (up to 100 $\mu$m) and distinctively complex morphology (Figure 6a and b); this closely resembled that previously described for osteoclasts (Chambers *et al.*, 1984). They had many fine microvilli on their free (upper) surface; these were usually concentrated over the central area of the cell which was often raised into a dome. OMGCs had lobulated and folded pseudopods over

**Figure 5** OMGC incubated for 30 min in medium containing CT (1 ng ml$^{-1}$) showing pale cytoplasm with broad pseudopods (×235).

at least part of their periphery, the cell margin at other points having fine filopodia or retraction fibres. The latter were buried in the bone surface or amongst the collagen fibres of a resorption cavity. No characteristic change in the morphological appearance of OMGCs or other cells was seen after OMGCs had been incubated in the presence of CT, PTH, 1,25 $(OH)_2$ $D_3$ or $PGE_2$ on SEM. The number of OMGCs present on each bone slice declined over the period of incubation in long-term cultures (Table II).

### *Bone resorption by OMGCs*

In all long-term cell cultures, the bone showed characteristic resorption pits in proximity to OMGCs. These closely resembled osteoclastic resorption pits, being sharply defined with coarsely fibrillar excavated bases. Frequently such pits were partly covered by an OMGC, the fine retraction fibres of the OMGC interdigitating with the mineralised collagen fibrils of the cavity base (Figure 6a and b). OMGCs produced resorption cavities of circular or serpiginous outlines and compound excavations which were combinations of the above types. In addition, poorly defined areas of discernible but unmeasurable roughening of the bone surface were also seen particularly around defined resorption pits. OMGCs were occasionally associated with more than one resorption pit separated by an intervening tract of undisturbed bone (Figure 6a). This appearance suggested that a single OMGC had produced these resorption pits and that bone resorption had been intermittent and not continuous as in the larger serpiginous or compound excavations. OMGCs also appeared to be capable of resorbing bone without the aid of other cells as resorption pits were often formed by OMGCs with no other cells in the vicinity, particularly in 24 h cultures (Figure 6a and b). OMGCs also resorbed anorganic bone in 24 h and 3 day cultures, indicating that they are capable of resorbing the mineral component of bone alone. The number of resorption pits on these anorganic bone slices corresponded closely with the number on untreated bone slices. Occasional smaller cells (less than 25 $\mu$m) were also associated with resorption pits (Figure 6d), suggesting that bone resorption was not only being carried out by large multinucleate cells but also by smaller possibly mononuclear cells.

Using two sets of paired bone slices, one of which was Triton-treated to remove the cells covering the bone surface, it was possible to determine changes in OMGC numbers and resorption cavity formation over time (Table II). Clearly, only a small minority of OMGCs were involved in bone resorption in control and hormone-treated cultures. The number of resorption pits did increase with the duration of incubation of cell cultures, indicating that OMGCs were capable of resorbing bone even after a prolonged period of incubation; this was also suggested by the occasional observation of OMGCs partially overlying a resorption pit in 3 day and 7 day cultures.

Bone resorption by OMGCs was seen in both control and hormone-treated cultures. CT had no effect on bone resorption by OMGCs; this was confirmed by measuring the number and area of these pits which did not differ significantly from control values. $PGE_2$ and 1,25-$(OH_2)$ vit $D_3$, known stimulators of bone resorption *in vivo*, also did not greatly influence the number or area of resorption pits produced by OMGCs. However, administration of PTH to OMGCs cultured on bone greatly increased the number of resorption pits (up to 20-fold). This effect was due to the hormone and not to differences in OMGC number seeded on the bones. The difference between PTH-treated and control or other hormone treated cultures was less marked at 3 days than 24 h, suggesting that PTH exerted its maximum influence on bone resorption in the first 24 h. The depth and surface area of the pits was not significantly influenced by hormone treatment although the largest and deepest pits on bone slices were present in PTH-treated cultures.

**Figure 6** (**a**) OMGC overlying simple resorption pit. Note broad pseudopods and retraction fibres at cell margin and microvilli over free surface (bar is 10 μm). (**b**) OMGC overlying complex compound resorption pit (bar is 10 μm). (**c**) Three simple resorption pits of roughly similar dimensions with intervening undisturbed bone (Triton-treated bone slice; bar is 10 μm). (**d**) Serpiginous resorption pit associated with a smaller cell of macrophage-like morphology (bar is 10 μm).

**Table II** Mean number of giant cells and resorption pits per bone slice with mean surface area of pits after 24 h and 3 days incubation on bone slices

| | *Mean number of giant cells per bone slice* | *Mean number of resorption pits (± s.e.m.)* | *Mean surface area of resorption pits ($\mu m^2$ ± s.e.m.)* |
|---|---|---|---|
| *24 h* | | | |
| Control | 54 | 2(±0.57) | 558 (±219) |
| PTH | 58 | 19 (±6.12)[a] | 653 (±76) |
| 1.25 $(OH)_2D_3$ | 62 | 1 (±0.33) | 277 (±117) |
| $PGE_2$ | 74 | 2 (±1.15) | 393 (±63) |
| Calcitonin | 50 | 1 (±0.57) | 421 (±127) |
| *3 days* | | | |
| Control | 28 | 2.30 (±1.85) | 424 (±125) |
| PTH | 26 | 25.67 (±10.01)[a] | 560 (±78) |
| 1.25 $(OH)_2D_3$ | 32 | 4.67 (±1.86) | 397 (±103) |
| $PGE_2$ | 35 | 3.0 (±1.53) | 283 (±48) |

[a]$P<0.05$ compared with controls, Student's $t$ test.

### Discussion

The superficial histological resemblance between osteoclasts and stromal giant cells found in several tumours in a number of extraskeletal sites has led to these giant cells being termed osteoclast-like. In this study, we have shown that a notable additional property which OMGCs isolated from a breast cancer share with osteoclasts is the ability to resorb bone; indeed, to our knowledge, this is the first direct demonstration of bone resorption by a cell type which has not been isolated from bone. In terms of morphological configuration and area, the well-defined areas of bone resorption described in association with OMGCs closely resemble those produced by osteoclasts (Athanasou *et al.*, 1984; Chambers *et al.*, 1984). The ability of OMGCs to resorb anorganic bone and the occasional observation of isolated OMGCs in close association with a resorption pit also suggests that OMGCs, like osteoclasts, are capable of resorbing all the components of bone. However, bone resorption by OMGCs significantly differs from that of isolated osteoclasts in that it is directly stimulated by PTH and not inhibited by calcitonin (Chambers *et al.*, 1984; McSheehy & Chambers, 1986*a,b*). PTH stimulation of osteoclastic bone resorption has only previously been seen when osteoclasts are incubated in the presence of osteoblasts, and osteoclastic bone resorption is also directly inhibited by calcitonin, a feature which is reflected by the characteristic change in the shape of the cell (Chambers & Magnus, 1982). This morphological response to calcitonin was not exhibited by OMGCs isolated in short-term cultures on coverslips.

Investigation of the antigenic phenotype of OMGCs also showed some similarities and differences between OMGCs and osteoclasts. OMGCs contained several antigenic determinants known to be present on osteoclasts including LCA (Athanasou *et al.*, 1987; Brecher *et al.*, 1986), CD13 and other macrophage-associated antigens (Athanasou *et al.*,

1988*a*), and platelet antigens glycoprotein IIIa and CD9 (Athanasou *et al.*, 1988*b*). However, OMGCs, unlike osteoclasts, were positive for HLA-DR, and Fc receptors as well as CD14, CD16, CD18 and CD11c (p150,95) antigens. All the above antigens are also expressed by mononuclear phagocytes (Hogg & Horton, 1987), indicating that OMGCs phenotypically can be regarded as a type of macrophage polykaryon. It should be noted, however, that OMGCs did not react with all the antimacrophage antibodies employed in this study. In addition, OMGCs like osteoclasts were positive for tartrate-resistant acid phosphatase. However, this enzyme cannot be regarded as a specific marker for osteoclasts (Andersson *et al.*, 1986). The absence of reaction for epithelial membrane antigen and cytokeratin staining is further proof that the OMGCs are unlike the epithelial tumour cells themselves.

Although osteoclasts and OMGCs were morphologically and functionally indistinguishable by scanning electron microscopy, OMGCs on transmission EM did not possess a complex ruffled border with a surrounding clear zone, the characteristic ultrastructural feature of the osteoclast (Gothlin & Ericsson, 1976). In addition, the numerous polyribosomes and cisternae of rER and the fine filament bundles present in the sub-plasmalemmal zone are not characteristic of osteoclasts and are more in keeping with the ultrastructure of a macrophage polykaryon (Papadimitriou & Walters, 1979).

The above findings suggest that OMGCs are a specific type of macrophage polykaryon which is distinct from both osteoclasts and other types of inflammatory polykaryons such as foreign-body giant cells. Unlike other macrophage polykaryons or macrophages themselves (Chambers & Horton, 1984), OMGCs can resorb bone in a similar manner to osteoclasts. Osteoclasts, like macrophage polykaryons (Sutton & Weiss, 1966; Chambers, 1978) are formed by the fusion of mononuclear precursors of bone marrow origin (Marks, 1983; Chambers, 1985) and there is now considerable evidence to suggest that these mononuclear precursors and osteoclasts themselves form part of the heterogeneous population of cells in the mononuclear phagocyte system (Mundy & Roodman, 1987). Accordingly, the cellular and functional differences noted between OMGCs, osteoclasts and macrophage polykaryons could be accounted for by the concept of macrophage heterogeneity (Hopper *et al.*, 1979). Moreover, the fact that OMGCs isolated from the breast are capable of resorbing bone indicates that a cell of the mononuclear phagocyte system, when transplanted to a different tissue, is capable of performing a highly specialised function of a cell of the mononuclear phagocyte system appropriate to that new tissue location. This suggests that the origin and diversity of macrophage heterogeneity may be determined by the particular local factors and tissue location in which macrophages find themselves (Metcalf, 1984).

*In vitro* transformation of peripheral blood monocytes to multinucleated giant cells is greatly increased in breast cancer patients (Al Sumidiae, 1986) and in healthy women with a strong family history of breast cancer (Morton *et al.*, 1988). It has been suggested that this type of giant cell formation is induced by a virus present in monocytes of breast cancer patients, and a retrovirus-like particle has recently been observed ultrastructurally in monocytes and cultured giant cells from patients with breast cancer (Al Sumidiae *et al.*, 1988). It is possible that a viral gene may also be present in that subpopulation of mononuclear phagocytes which form OMGCs in breast cancer with numerous OMGCs. Viral genes could account for the distinct functional and antigenic differences of these cells from tissue macrophages, inflammatory polykarya and osteoclasts. However, it should be noted that we did not observe virus-like particles in stromal mononuclear cells or OMGCs such as those reported above. Nor have they been reported in any of the many previous ultrastructural studies of breast carcinoma with OMGCs (Factor *et al.*, 1977; Agnantis & Rosen, 1978; Rosen, 1979; Holland & van Haelst, 1983; Sugano *et al.*, 1983; Nielsen & Kiaer, 1985; Tavassoli & Norris, 1986; McMahon *et al.*, 1986; Gupta *et al.*, 1987).

The existence of an osteoclast-like cell which is directly stimulated by PTH to resorb bone may provide a clue to the cellular and molecular mechanisms of osteolysis associated with malignant tumours. Osteoclasts are generally regarded as the principal cell responsible for bone destruction in solid tumours both in the presence and absence of metastases in bone. No direct evidence of osteolysis by tumour cells has been presented to date (Chambers, 1985). Our observation of PTH stimulation of bone resorption by cells isolated from a breast carcinoma supports the belief that PTH or a PTH-like protein is important in the humoral hypercalcaemia of malignancy (Mundy *et al.*, 1984; Suva *et al.*, 1987). It also suggests a possible mechanism whereby solid tumours, such as breast cancer, with bone metastases can effect bone resorption. One of the known effects of PTH is to increase the number of osteoclasts formed from pre-existing precursors (Wong, 1986). Production of a PTH-like protein by breast cancer cells may be responsible for the accumulation of OMGCs in the unusual primary breast cancer studied. These OMGCs, although capable of bone resorption, have no substrate to resorb in the breast. However, in the more common situation of metastatic breast cancer in bone, secretion of a PTH-like protein by cancer cells in bone would lead to a similar accumulation of OMGCs and these would then resorb the bony substrate in the vicinity of the metastatic tumour cells.

The normal plasma levels of calcium and phosphate seen in the case described are consistent with this concept. In addition, the finding of smaller, possibly mononuclear, cells in association with resorption pits suggests that mononuclear phagocytes, which are commonly found in the inflammatory infiltrate around malignant tumours, may be similarly stimulated and capable of bone resorption.

This work has been funded by the Arthritis and Rheumatism Council. We would like to thank Miss L. Watts for typing the manuscript and Mr G. Richardson for processing the photographs.

### References

AGNANTIS, N.T. & ROSEN, P.P. (1978). Mammary carcinoma with osteoclast-like giant cells. A study of eight cases with follow-up data. *Am. J. Clin. Pathol.*, **72**, 383.

AL SUMIDIAE, A.M., LEINSTER, S.J. & JENKINS, S.A. (1986). Transformation of blood monocytes to giant cells *in vitro* from patients with breast cancer. *Br. J. Surg.*, **73**, 839.

AL SUMIDIAE, A.M., LEINSTER, S.J., HART, C.A., GREEN, C.D. & McCARTHY, K. (1988). Particles with properties of retroviruses in monocytes from patients with breast cancer. *Lancet*, **i**, 5.

ANDERSSON, G.N., EK-RYLANDER, B., HAMMARSTROM, L.E., LINDSKOG, S. & TOVERVAL, S. (1986). Immunocytochemical localization of a tartrate-resistant and varadate sensitive acid nucleotide trio and diphosphatase. *J. Histochem. Cytochem.*, **34**, 293.

ANDREEV, V.C., RAITCHEV, R. & NIKOLOVA, D. (1964). Malignant osteoclastoma of the skin. *Br. J. Dermatol.*, **76**, 40.

ATHANASOU, N.A., GRAY, A., REVELL, D.A., FULLER, K., COCHRANE, T. & CHAMBERS, T.J. (1984). Stereophotogrammetric observations on bone resorption by isolated rabbit osteoclasts. *Micron. Microscop. Acta.*, **15**, 47.

ATHANASOU, N.A., QUINN, J. & McGEE, J.O'D. (1987). Leucocyte common antigen is present on osteoclasts. *J. Pathol.*, **153**, 121.

ATHANASOU, N.A., QUINN, J. McGEE, J.O'D. (1988*a*). Immunocytochemical analysis of the human osteoclast: phenotypic relationship to other marrow-derived cells. *Bone Mineral*, **3**, 317.

ATHANASOU, N.A., QUINN, J., HERYET, A. & McGEE, J.O'D. (1988*b*). Localization of platelet antigens and fibrinogen on osteoclasts. *J. Cell Sci.*, **89**, 115.

BALOGH, K., WOLBARSHT, R.L., FEDERMAN, M. & O'HARA, C.J. (1985). Carcinoma of the parotid gland with osteoclast-like giant cells. *Arch. Pathol. Lab. Med.*, **109**, 756.

BERENDT, R.C., SHNITKA, T.K., WIENS, E., MANICKAVEL, V. & JEWELL, L.D. (1987). The osteoclast-type giant cell tumor of the pancreas. *Arch. Pathol. Lab. Med.*, **111**, 43.

BRECHER, M., FRANKLIN, W.A. & SIMON, M.A. (1986). Immunohistochemical study of mononuclear phagocyte antigens in giant cell tumor of bone. *Am. J. Pathol.*, **125**, 252.

CHAMBERS, T.J. (1978). Multinucleate giant cells. *J. Pathol.*, **126**, 125.

CHAMBERS, T.J. (1985). The pathobiology of the osteoclast. *J. Clin. Pathol.*, **38**, 241.

CHAMBERS, T.J. & HORTON, M.A. (1984). Failure of cells of the mononuclear-phagocyte series to resorb bone. *Calcif. Tissue Int.*, **36**, 556.

CHAMBERS, T.J. & MAGNUS, C.J. (1982). Calcitonin alters behaviour of isolated osteoclasts. *J. Pathol.*, **136**, 27.

CHAMBERS, T.J., REVELL, P.A., FULLER, K. & ATHANASOU, N.A. (1984). Resorption of bone by isolated rabbit osteoclasts. *J. Cell Sci.*, **66**, 383.

CORDELL, J., RICHARDSON, T.C., PULFORD, K.A.F. & 4 others (1985). Production of monoclonal antibodies against human epithelial membrane antigen for use in diagnostic immunocytochemistry. *Br. J. Cancer*, **52**, 347.

DORNEY, P. (1967). Osteoclastoma of the heart. *Br. Heart J.*, **29**, 276.

ESHUN-WILSON, K. (1973). Malignant giant cell tumour of the colon. *Acta Pathol. Microbiol. Scand.*, **81**, 137.

EVANS, R.A., DUNSTAN, C.R. & BAYLINK, D.J. (1979). Histochemical identification of osteoclasts in undecalcified sections of human bone. *Mineral Electrolyte Metabol.*, **2**, 179.

FACTOR, S.M., BIEMPICA, L., RATNER, I., AHUJA, K.K. & BIEMPICA, S. (1977). Carcinoma of the breast with multinucleated reactive stromal giant cells. A light and electron microscopic study of two cases. *Virchows Archiv A Path. Anat. Histol.*, **374**, 1.

FRY, H.J.B. (1927). Osteoclastoma (myeloid sarcoma) of the human female breast. *J. Pathol. Bacteriol.*, **30**, 529.

GATTER, K.C., FALINI, B. & MASON, D.Y. (1984). The use of monoclonal antibodies in histopathological diagnosis. In *Recent Advances in Histopathology, No. 12*, Anthony, P.P. & MacSween, R.N.M. (eds) p. 35. Churchill Livingstone: Edinburgh.

GOTHLIN, G. & ERICSSON, J.L.E. (1976). The osteoclast. *Clin. Orthop.*, **120**, 201.

GUPTA, R.K., ST JOHN, W., HOLLOWAY, L.J. & SIMPSON, J.S. (1988). Immunocytochemical and ultrastructural study of the rare osteoclast-type carcinoma of the breast in a fine needle aspirate. *Acta Cytol.*, **32**, 179.

HOGG, N. & HORTON, M.A. (1987). Myeloid antigens: new and previously defined clusters. In *Leucocyte Typing III*, McMichael, A. *et al.* (eds) p. 576. Oxford University Press: Oxford.

HOLLAND, R. & VAN HAELST, U.J.G.M. (1984). Mammary carcinoma with osteoclast-like giant cells. Additional observations on six cases. *Cancer*, **53**, 1963.

HOPPER, K.E., WOOD, P.R. & NELSON, D.S. (1979). Macrophage heterogeneity. *Vox Sang.*, **36**, 257.

KELLY, P.M.A., BLISS, E., MORTON, J., BURNS, J. & MCGEE, J.O'D. (1988). Monoclonal antibody EBM/11: high cellular specificity for human macrophages. *J. Clin. Pathol.*, **41**, 510.

MACDONALD, S.M., PULFORD, K., FALINI, B., MICKLEM, K. & MASON, D.Y. (1986). A monoclonal antibody recognizing the p150/95 leucocyte differentiation antigen. *Immunology*, **59**, 427.

MAKIN, C.A., BOBROW, L.G. & BODMER, W.F. (1984). Monoclonal antibody to cytokeratin for routine use in routine histopathology. *J. Clin. Pathol.*, **34**, 975.

MARKS, S.C. (1983). The origin of osteoclasts. *J. Oral Pathol.*, **12**, 226.

MCMAHON, R.F.T., AHMED, A. & CONNOLLY, C.E. (1986). Breast carcinoma with stromal multinucleated giant cells – a light microscopic, histochemical and ultrastructural study. *J. Pathol.*, **150**, 175.

MCSHEEHY, P.M.J. & CHAMBERS, T.J. (1986*a*). Osteoblastic cells mediate osteoclastic responsiveness to parathyroid hormone. *Endocrinology*, **118**, 824.

MCSHEEHY, P.M.J. & CHAMBERS, T.J. (1986*b*). Osteoblast-like cells in the presence of parathyroid hormone release soluble factor that stimulates osteoclastic bone resorption. *Endocrinology*, **119**, 1654.

METCALF, D. (1984). Macrophage heterogeneity. In *Mononuclear Phagocyte Biology*, Volkman, A. (ed) p. 489. Marcel Dekker: New York.

MORTON, A.R., TESTA, N.G., BURNS, P., BIRCH, J. & HOWELL, A. (1988). Peripheral blood mononuclear cells from relatives of women with hereditary breast cancer form multinucleate giant cells *in vitro*. *Calcif. Tissue Int.*, **42** (suppl), A44.

MOSELEY, J.M., KUBOTA, M., DIEFERBACK-JAGGER, H., WETTENHALL, R.E.H., KEMP, B.E. *et al.* (1987). Parathyroid hormone-related protein purified from a human lung cancer cell line. *Proc. Natl Acad. Sci. USA*, **84**, 5048.

MUNDY, G.R., IBBOTSON, K.J., D'SOUZA, S.M., SIMPSON, E.L., JACOBS, J.W. & MARTIN, T.J. (1984). The hypercalcaemia of cancer: clinical implications and pathogenic mechanisms. *N. Engl. J. Med.*, **310**, 1718.

MUNDY, G.R. & ROODMAN, G.D. (1987). Osteoclast ontogeny and function. In *Bone and Mineral Research No. 5*, Peck, W.A. (ed) p. 209. Elsevier: Amsterdam.

NAIEM, M., GERDES, J., ABDULAZIZ, Z., NASH, J., STEIN, H. & MASON, D.Y. (1981). Production of monoclonal antibodies for the immunohistological diagnosis of human lymphoma. In *Leukaemia Markers*, Knapp, W. *et al.* (eds) p. 117. Academic Press: New York.

NIELSEN, B.B. & KIAER, H.W. (1985). Carcinoma of the breast with stromal multinucleated giant cells. *Histopathology*, **9**, 183.

NISHIYAMA, R.H., DUNN, E.L. & THOMPSON, N.W. (1972). Anaplastic spindle cell and giant cell tumors of the thyroid gland. *Cancer*, **30**, 113.

PAPADIMITRIOU, J.M. & WALTERS, M.N-I. (1979). Macrophage polykarya. *CRC Crit. Rev. Toxicol.*, **6**, 221.

ROSEN, P.P. (1979). Multinucleated mammary stromal giant cells. *Cancer*, **44**, 1305.

SUGANO, I., NAGAO, K., KONDO, Y., NABESHIMA, S. & MURAKAMI, S. (1983). Cytologic and ultrastructural studies of a rare breast carcinoma with osteoclast-like giant cells. *Cancer*, **52**, 74.

SUTTON, J.S. & WEISS, L. (1966). Transformation of monocytes in tissue culture into macrophages, epitheliod cells, and multinucleated giant cells: an electron microscope study. *J. Cell Biol.*, **28**, 303.

SUVA, L.J., WINSLOW, G.A., WETTENHALL, R.E.H., HAMMOND, R.G., MOSELEY, J.M. *et al.* (1987). A parathyroid hormone-related protein implicated in malignant hypercalcaemia: cloning and expression. *Science*, **237**, 893.

TAVASSOLI, F.A. & NORRIS, H.J. (1986). Breast carcinoma with osteoclast-like giant cells. *Arch. Pathol. Lab. Med.*, **110**, 636.

TERMINE, J.D., EANES, E.D., GREENFIELD, D.J., NYLER, M.V. & HARPER, R.A. (1973). Hydrazine-deproteinated bone mineral. *Calcif. Tissue Res.*, **12**, 73.

TETTEROO, P.A.T., LANDSDORP, P.M., LEEKSMA, O.C. & VON DEN BORNE, A.F.G. (1983). Monoclonal antibodies against human glycoprotein IIIa. *Br. J. Haematol.*, **55**, 509.

VIAC, J., REANO, A., BROCHIER, J. *et al.* (1981). Reactivity pattern of a monoclonal antikeratin antibody (KL1). *J. Invest. Dermatol.*, **81**, 351.

WARNKE, R., GATTER, K.C., FALINI, B. *et al.* (1983). Diagnosis of human lymphoma with monoclonal antileukocyte antibodies. *N. Engl. J. Med.*, **309**, 1275.

WONG, G.L. (1986). Skeletal effects of parathyroid hormone. In *Bone and Mineral Research, No. 4*, Peck, W.A. (ed) p. 103. Elsevier: Amsterdam.